# Historical Assessment and Mapping of Human Plague, Kazakhstan, 1926–2003

**DOI:** 10.3201/eid3012.231659

**Published:** 2024-12

**Authors:** Nurkuisa Rametov, Ziyat Abdel, Zauresh Zhumadilova, Duman Yessimseit, Beck Abdeliyev, Raikhan Mussagaliyeva, Svetlana Issaeva, Omar F. Althuwaynee, Zhaksybek Baygurin, Kairat Tabynov

**Affiliations:** Institute of Ionosphere, Almaty, Kazakhstan (N. Rametov); M. Aikimbayev National Scientific Center for Especially Dangerous Infections, Almaty (N. Rametov, Z. Abdel, Z. Zhumadilova, D. Yessimseit, B. Abdeliyev, R. Mussagaliyeva, S. Issaeva, K. Tabynov); Kazakh National Research Technical University named after K.I. Satbayev, Almaty (N. Rametov, Z. Baygurin); Institute for Geo-Hydrological Protection of the Italian National Research Council, Perugia, Italy (O.F. Althuwaynee); Kazakh National Agrarian Research University, Almaty (K. Tabynov)

**Keywords:** Plague, Yersinia pestis, zoonoses, vector-borne infections, bacteria, epidemiology, mapping, healthcare, outbreaks, Kazakhstan

## Abstract

Understanding Kazakhstan’s plague history is crucial for early warning and effective health disaster management. We used descriptive-analytical methods to analyze spatial data for human cases in natural plague foci in Kazakhstan during 1926–2003. The findings revealed 565 human cases across 82 outbreaks in Almaty (32.22%), Aktobe (1.59%), Atyrau (4.42%), Mangystau (21.24%), and Kyzylorda (40.53%) oblasts. Before antibiotic drugs were introduced in 1947–1948, major plague outbreaks occurred in 1926, 1929, 1945, 1947, and 1948, constituting 80.7% of human transmission. Plague spread through flea bites, camel handling, wild animal contact, aerosol transmissions, and rodent bites. Patients were up to 86 years of age; 49.9% were male and 50.1% female. Pulmonary cases were reported most frequently (72.4%), and person-to-person infection occurred at an incidence rate of 0.29 cases/10,000 population. Risk increased with human expansion into natural plague foci areas. Swift diagnosis and treatment are essential for curbing plague outbreaks in Kazakhstan.

The Soviet plague control system was established in the early 20th Century as a comprehensive strategy to combat plague outbreaks across the vast territories of the former Soviet Union. That system was characterized by its focus on surveillance, prevention, and rapid response to plague epidemics. The approach involved the creation of specialized plague control institutes and stations, which were responsible for monitoring and controlling the spread of plague within designated areas. In Kazakhstan, which has a long history of plague endemicity, the Soviet system was adapted to suit the region’s unique geographic and ecologic conditions. Soviet authorities divided plague-endemic territories into natural foci on the basis of ecologic and epidemiologic characteristics. Within those larger foci, the territory was further subdivided into smaller subfoci that represented more specific areas where plague activity was particularly concentrated, often because of specific ecologic conditions, such as soil composition, climate, and particular rodent and flea species.

Plague is an endemic disease of Kazakhstan, and epidemic outbreaks of plague among the population have been known for a long time (*1*–*3*), which is evidenced by the historical names of geographic areas that include the Kazakh root *oba* (plague), including Karaoba (*kara* [black] plus *oba*); Kosoba (*kos* [both] plus *oba*); Kyzyloba (*kyzyl* [red] plus *oba*), and Besoba (*bes* [five] plus *oba*) (*1*,*2*). In the late 19th and early 20th Centuries, outbreaks of plague among the population of western Kazakhstan became more frequent and larger. During 1905–1906, an epidemic of the so-called Beketayev plague was registered in the Naryn part of the Volga-Ural sands, where 659 persons fell ill and 621 died (*1*–*5*). Later, outbreaks of plague among the population of Kazakhstan were also noted in 1907, 1910–1914, 1918, and 1928. However, plague epidemics did not begin to be documented by descriptive epidemiology and microbiology until 1913 (*3*–*5*). We used historical data on plague recorded in Kazakhstan to describe the epidemiology and spatial characteristics of human plague cases during 1926–2003.

## Methods

### Terminology

In the context of plague epidemiology, the term natural plague foci (NPF) refers to relatively limited geographic areas where the plague-causing bacterium, *Yersinia pestis*, circulates persistently among wildlife, particularly rodent populations, over extended periods. Those areas are characterized by specific ecologic conditions that support the continuous presence of the bacterium, its hosts, and vectors, leading to the long-term maintenance of the disease in the environment.

Autonomous plague foci refers to specific geographic areas where *Y. pestis* circulates and persists independently over time without relying on external sources of infection. Those foci are characterized by a stable ecologic and epidemiologic environment that supports *Y. pestis* persistence and transmission among local wildlife, particularly rodent populations and flea vectors. In autonomous plague foci, the disease cycle is self-sustaining, meaning that *Y. pestis* can be maintained within the local ecosystem for extended periods, often decades or even centuries, without the need for reintroduction from other regions. Those foci are essential for understanding the long-term dynamics of plague and are critical targets for surveillance and control efforts to prevent the spread of the disease to surrounding areas.

The term plague carriers refers to persons who were asymptomatic but tested positive for *Y. pestis* through laboratory confirmation. Thus, those persons carried the bacterium without exhibiting typical plague symptoms.

### Criteria for Defining Foci and Subfoci

We used the following criteria for defining foci and subfoci. First was, geographic boundaries, the natural boundaries of the landscape, such as mountains, rivers, and deserts, that influenced the distribution of rodent populations. Second was ecologic conditions, which include the type of vegetation, soil characteristics, and climate that affect the habitat suitability for *Y. pestis* reservoirs and vectors. Third was rodent and flea populations and density of specific rodent species known to be primary hosts for *Y. pestis* and the flea species that serve as vectors. Finally was historical data, including records of plague outbreaks and epizootics in the region, which helped identify areas with persistent plague activity.

Adaptation of the Soviet plague control system in Kazakhstan led to the systematic categorization of the region’s plague-endemic areas into foci and subfoci, which were critical for targeted surveillance and control measures. That framework remains a cornerstone of plague management in the region, guiding current efforts to monitor and mitigate risk for outbreaks.

### Study Area

**Figure 1:**
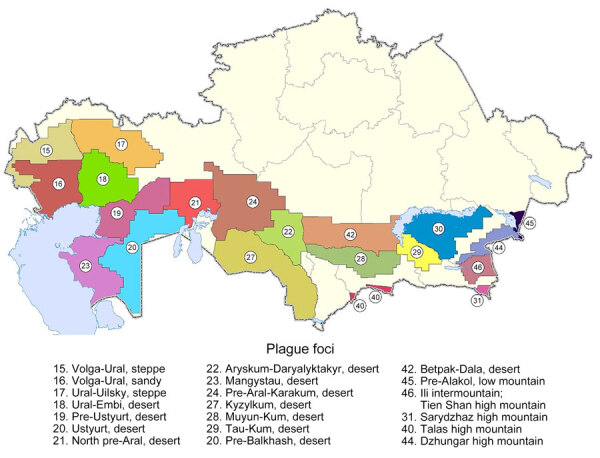
Locations of natural plague foci in a historical assessment and mapping of human plague, Kazakhstan, 1926–2003. Codes according to the unified country certification system, Commonwealth of Independent States. Codes indicate location names and terrain type.

The natural plague endemic range in Kazakhstan is located on an area of 1,117,000 km^2^, which is ≈41.0% of the country. The republic includes 6 natural and 15 autonomous plague foci, within which >90 landscape-epizootologic districts are allocated (*6*,*7*) (Figure 1).

The main carriers of plague in the region are gerbils (*Rhombomys opimus*), ground squirrels (*Spermophilus* spp.), and marmots (*Marmota* spp.); the main flea vectors are in the genera *Xenopsylla* and *Nosopsyllus*. In Kazakhstan, plague was found in >40 species of rodents, carnivorous and insectivorous mammals, lagomorphs, ungulates, and 2 species of birds (*5*,*6*,*8*).

### Human Plague Cases

We collected records of human plague cases in Kazakhstan from 1926 through 2003 from literature sources (*3*–*6*,*8*–*11*). Those sources identified each case by its history, site, disease outcome, suspected source of infection, clinical form, clinical confirmation, and laboratory confirmation through bacteriology or serology. We calculated incidence per 10,000 population. We collected data on the population during 1926–2003 from the archive of demographic documents (*12*–*14*).

**Figure 2:**
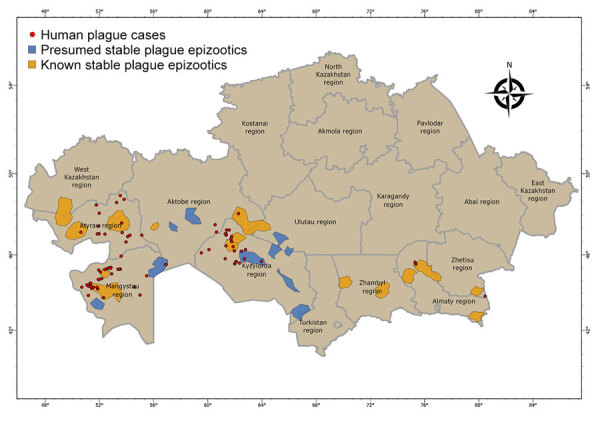
Epizootologic zoning of plague in a historical assessment and mapping of human plague, Kazakhstan, 1926–2003. Human plague cases and known and presumed plague epizootic areas are noted.

We determined geographic coordinates of human plague sites by using literature sources that included an approximate area of 1,007,350 km^2^ (*3*–*6*,*8*–*10*,*15*). We used ArcGIS Pro 2.7 software (ESRI, https://www.arcgis.com; *15*) to digitize and map locations of plague cases (Figure 2).

### Statistical Analysis

We used GraphPad Prism software version 9.0.0 (GraphPad Software Inc., https://www.graphpad.com) to perform statistical analysis. To analyze age and gender differences between groups, we used 2-way analysis of variance, then the Sidak or Tukey multiple comparisons test. We considered p<0.05 statistically significant.

## Results

### Human Plague Cases

During 1926–2003, a total of 565 plague cases, including 2 cases of plague carriers, were registered in 82 foci (*3*–*6*,*8*–*10*,*15*). We included those cases in the analysis of epidemiology of plague in Kazakhstan. The last known case occurred in 2003. Plague outbreaks have been registered in 10 of 20 natural plague foci (*3*–*6*,*8*) (Table 1).

**Table 1:** Natural plague foci and years of registration of human plague cases in a historical assessment and mapping of human plague, Kazakhstan, 1926–2003

Region	Natural plague foci	Main rodent host	Main flea vector	Year of human plague cases
Atyrau	Volga-Ural Sand	*Meriones meridianus*, *M. tamariscinus*	*Xenopsylla conformis*, *Nosopsyllus laeviceps*	1997
Atyrau	Ural-Emba	*Rhombomys opimus*	*Xenopsylla skrjabini*, *X. conformis*, *N. laeviceps*, *Coptopsylla lamellifer*	1956, 1958, 1964, 1968, 1986, 1988, 1989, 1990, 1992, 1993
Atyrau, Mangystau	Pre-Ustyurt	*R. opimus*	*X. skrjabini*, *N. laeviceps*	1958, 1959, 1961, 1967, 1975
Mangystau, Aktobe	Ustyurt	*R. opimus*	*X. skrjabini*, *Xenopsylla nuttalli*, *Xenopsylla gerbilli*	1926, 1974, 1975, 1999
Aktobe, Kyzylorda	North Pre-Aral	*R. opimus*	*X. skrjabini*, *N.laeviceps*, *C. lamellifer*	1945,1993, 1999, 2002
Mangystau	Mangyshlak	*R. opimus*	*X. skrjabini*, *Xenopsylla nuttalli*	1926, 1927, 1948, 1964, 1973, 1974, 2003
Aktobe, Kyzylorda	Pre–Aral-Karakum	*R. opimus*	*X. skrjabini*, *N. laeviceps*, *C. lamellifer*	1947, 1948, 1955, 1959, 1966, 1967, 1969, 1971, 1972, 1979, 1990, 1991, 1999, 2001, 2003
Kyzylorda	Kyzylkum	*R. opimus*	*X. skrjabini*, *X. gerbilli*, *Xenopsylla hirtipes*	1966, 1971,1993, 1999
Almaty	Pre-Balkhash	*R. opimus*	*X. skrjabini*, *X. gerbilli*, *X. hirtipes*	1947, 1948, 1989
Almaty	Ili Intermountain	*R. opimus*	*X. gerbilli*, *X. hirtipes*	1929

During the 1950s through the 1970s, mass field teams worked in Kazakhstan and identified areas with stable and suspected plague epizootics (*1*,*2*,*4*). Almost all studied human plague cases overlapped with areas of stable plague epizootics (*3*) (Figure 2).

**Figure 3:**
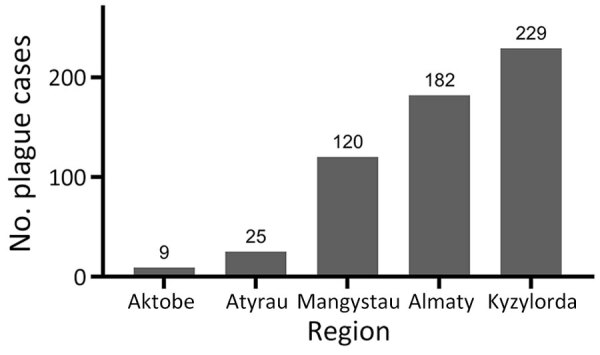
Numbers of human plague cases by region in a historical assessment and mapping of human plague, Kazakhstan, 1926–2003.

**Figure 4:**
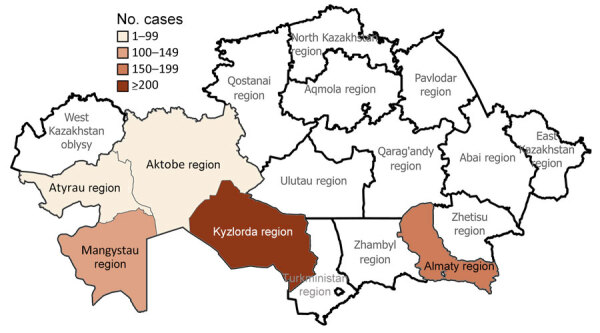
Locations of human plague cases by region in a historical assessment and mapping of human plague, Kazakhstan, 1926–2003.

During the study period, human plague cases were registered in the regions of Almaty (32.22% of all cases), Aktobe (1.59%), Atyrau (4.42%), Mangystau (21.24%), and Kyzylorda (40.53%). The incidence rate (IR) for all cases registered during the study period was 0.29/10,000 population. The IR per 10,000 population by region was 0.52 in Almaty, 0.05 in Aktobe and Atyrau, 1.11 in Mangystau, and 0.27 in Kyzylorda (Figures 3, 4).

**Figure 5:**
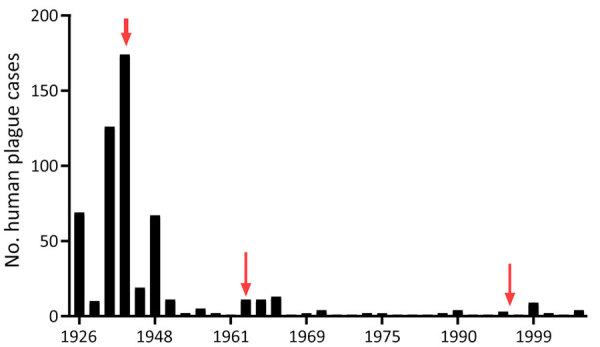
Annual number of cases in a historical assessment and mapping of human plague, Kazakhstan, 1926–2003. Red arrows indicate peaks.

At the beginning of the 20th Century, many plague cases were reported, and then cases decreased. We noted 3 plague peaks during the study period (Figure 5). The first peak occurred during 1926–1948 in the west and south, where 82% of all cases were reported. For example, in 1 outbreak in Kyzylorda oblast in 1945, more than one third (37.4%) of the population became ill with plague. Those epidemics required the organization of scientific and methodology centers in Central Asia. By the Order of the Ministry of Health of the USSR, No. 739, dated December 9, 1948, and January 1, 1949, the Almaty Anti-Plague Station was transformed into the Central Asian Research Anti-Plague Institute of the Ministry of Health of the USSR, later renamed M. Aikimbayev National Scientific Center for Especially Dangerous Infections (NSCEDI) of the Ministry of Health of the Republic of Kazakhstan. NSCEDI conducted research work on plague; produced diagnostic preparations; and provided scientific, methodological, and operational guidance on the organization and implementation of a set of sanitary and prophylactic measures for plague control stations in Kazakhstan and other Central Asia republics. In addition, during 1947–1948, the Public Health Service of Kazakhstan began to use antibiotic drugs to treat plague, vaccinate the population to prevent the disease, conduct sanitary and preventive work against plague, and conduct epidemiologic field studies. After those measures were applied, plague incidence rapidly decreased.

The second plague period occurred during 1955–1989, which accounted for 13.0% of all cases during the analyzed period. Few cases were reported during that time, but a slight increase was noted during 1961–1967 related to the slaughter of plague-affected camels. During those years, 6.37% of the population contracted plague, among whom 75.0% were infected through contact with sick camels. At one time, dozens of persons participated in the slaughter of camels, and all could have contracted plague at the same time. Then, the government issued an order stating that camel owners could receive compensation if their camels were infected with plague and the owners reported infected camels to the authorities. That order contributed to the reduction of cases involving sick camels (*1*,*3*,*4*).

A third period of increasing incidence occurred during 1990–2003, which accounted for 5.0% of all cases during the analyzed period. During that time, the Soviet Union collapsed, infrastructure was destroyed, and the economy in Kazakhstan deteriorated. Epidemiologic surveillance of plague was not fully funded and could not cover all plague endemic areas. After the economic situation of the country improved, plague incidence decreased, and the last case was registered in 2003 (Figure 5).

**Figure 6:**
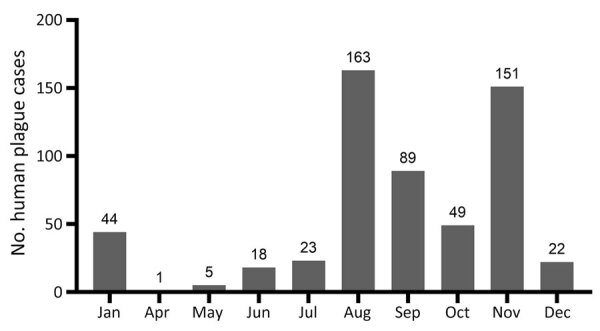
Human plague cases by month in a historical assessment and mapping of human plague, Kazakhstan, 1926–2003.

Most plague cases were reported in the months of January, August, September, October, and November (Figure 6). January, October, November, and December cases occurred during major outbreaks recorded in 1926, 1929, 1945, 1947, and 1948, and plague was spread by humans during those outbreaks (Figure 6).

During the study period, we noted multiple clinical forms of plague. Among reports we found 12.57% bubonic, 5.84% bubonic septicemic, 1.06% bubonic pneumonia, 72.4% pulmonary, 0.35% secondary pneumonia, 2.83% septicemic, 0.18% cutaneous, 0.88% cutaneous bubonic, 0.35% tonsillar, and 0.18% tonsillar bubonic forms; 3.36% of cases had no clinical form data. We also observed that 71.15% of plague infections occurred from person-to-person transmission. Other infection sources included fleas (12.39%), camels (12.39%), hares (0.88%), aerosols (0.53%), foxes (0.35%), rodent bites (0.18%), saiga antelope (*Saiga tatarica*) (0.18%), and feral cats (0.18%); 1.77% of cases had no available transmission data.

**Figure 7:**
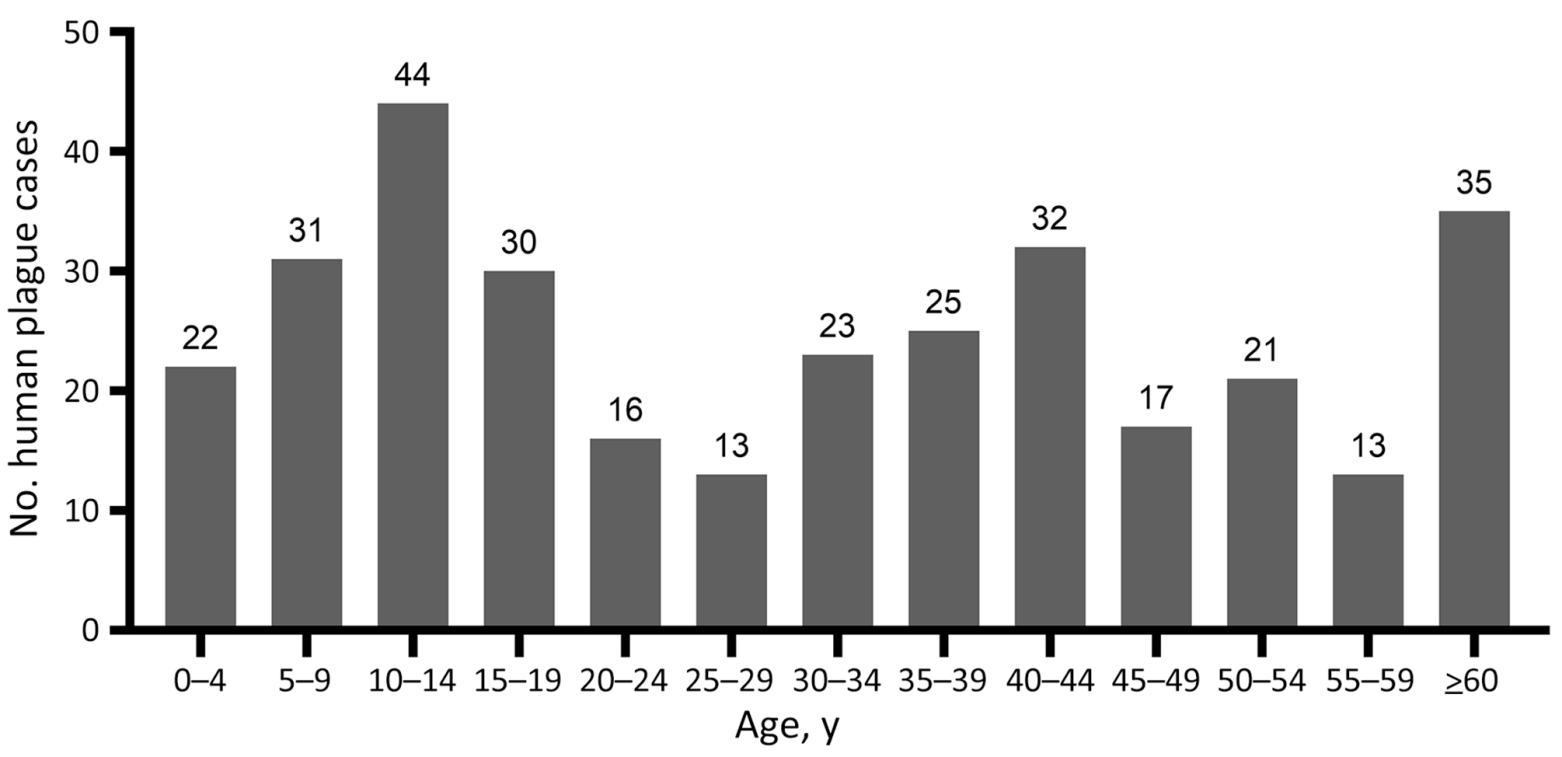
Human plague cases by age group a historical assessment and mapping of human plague, Kazakhstan, 1926–2003.

**Figure 8:**
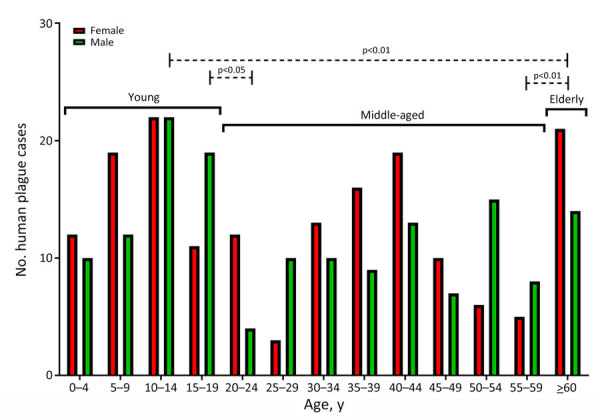
Human plague cases by age group and sex in a historical assessment and mapping of human plague, Kazakhstan, 1926–2003.

Among case-patients, we noted 3 age subgroups: young (0–19 years of age), middle (20–59 years of age), and older (>60 years of age) (Figure 7). When we analyzed age and sex distribution, we found that female and male persons were at equal risk for plague infection. However, we found age differences for both sexes in the young, middle, and older age groups (Figure 8). Most cases among female persons were registered in 8 different age groups. The highest number of cases for both sexes was observed in the 10–14-year age group. The highest number of cases in women was observed in the >60-year age group and for male persons in the 15–19-year age group. The lowest number of cases among women was observed in the 25–29-year age group and among men in the 20–24-year age group (Figure 8).

In 1913, human plague epidemics began to be documented by using descriptive epidemiology and microbiology (*12*). Cases were confirmed on the basis of epidemiologic, clinical, serologic, and bacteriologic data. Most human plague cases were confirmed by bacteriologic methods and isolation of *Y. pestis* (Table 2).

**Table 2:** Percentages of registered human plague cases per year in a historical assessment and mapping of human plague, Kazakhstan, 1926–2003*

Year	% Cases per region					% Total cases	% Confirmed (% not confirmed)	
	Aktobe	Almaty	Atyrau	Kyzylorda	Mangystau		Bacteriology	Serology
1926	0	0	0	0	12.2	12.2	100.0	–
1927	0	0	0	0	1.77	1.77	No data	No data
1929	0	22.3	0	0	0	22.3	100.0	–
1945	0	0	0	30.8	0	30.8	100.0	–
1947	0	2.3	0	1.06	0	3.36	100.0	–
1948	0	7.43	0	0.35	4.07	11.86	92.5 (7.5)‡	–
1955	0	0	0	1.95	0	1.95	100.0	–
1956	0	0	0.35	0	0	0.35	100.0	–
1958	0	0	0.88	0	0	0.88	20.0 (80.0)‡	–
1959	0	0	0.18	0.18	0	0.35	100.0	–
1961	0	0	0.18	0	0	0.18	100.0	–
1964	0	0	0.18	0	1.77	1.95	100.0	–
1966	0.35	0	0	1.59	0	1.95	36.36 (63.64)‡	–
1967	0	0	1.06	1.24	0	2.3	38.46 (46.15)‡	15.38
1968	0	0	0.18	0	0	0.18	100.0	–
1969	0	0	0	0.35	0	0.35	100.0	–
1971	0	0	0	0.71	0	0.71	No data	50.0
1972	0	0	0	0.18	0	0.18	100.0	–
1973	0	0	0	0	0.18	0.18	100.0	–
1974	0	0	0	0	0.35	0.35	100.0	–
1975	0	0	0	0	0.35	0.35	50.0 (50.0)‡	–
1979	0	0	0	0.18	0	0.18	100.0	–
1986	0	0	0.18	0	0	0.18	100.0	–
1988	0	0	0.18	0	0	0.18	100.0	–
1989	0	0.18	0.18	0	0	0.35	100.0	–
1990	0	0	0.35	0.35	0	0.71	75.0	25.0
1991	0	0	0	0.18	0	0.18	100.0	–
1992	0	0	0.18	0	0	0.18	100.0	–
1993	0.18	0	0.18	0.18	0	0.53	100.0	–
1997	0	0	0.18	0	0	0.18	100.0	–
1999	1.06†	0	0	0.53	0	1.59	100.0	–
2001	0	0	0	0.35	0	0.35	50.0	50.0
2002	0	0	0	0.18	0	0.18	0	100.0
2003	0	0	0	0.18	0.53	0.71	75.0	25.0
Total	1.59†	32.21	4.44	40.54	21.22	100.0	92.3	1.41

*­­–, cases that were not tested †Cases included 2 plague carrier cases. ‡*Y. pestis* not isolated.

**Figure 9:**
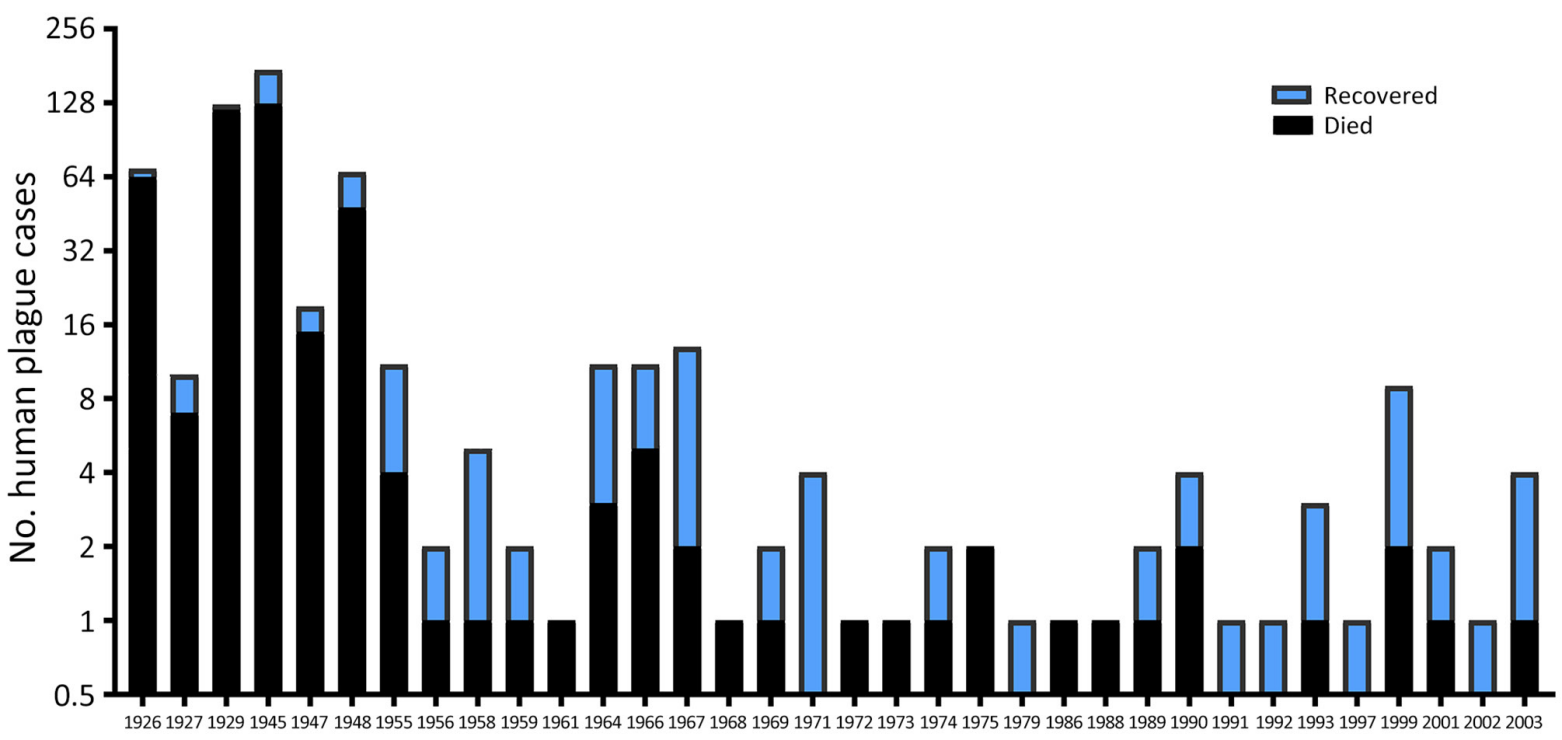
Human plague cases by outcome in a historical assessment and mapping of human plague, Kazakhstan, 1926–2003.

We noted that higher mortality rates were recorded in 1926, 1927, 1929, 1945, and 1948 (Figure 9). In total, ≈26% of patients recovered from plague. Areas with higher mortality rates included Mangystau oblast in 1926, 1927, and 1948; Alma-Ata in 1929 and 1948; and Kyzylorda oblast in 1945. 

During the study period, the IR was 0.01–56.1/10,000 population, and case-fatality rates (CFRs) ranged up to 100% (Table 3), but CFR was high for most of the study period. IR was high during 1926–1927 but declined after that timeframe (Table 3). The highest observed IR was from the Kul Kara settlement in Mangystau oblast in 1926. During that outbreak in mid-August, the death of a child led to person-to-person spread of infection. By September 20 of that year, 41 of 72 persons living in the village had died of pneumonic plague, and other cases were registered in different villages in Buzachi Peninsula of the oblast. At that time, Mangystau oblast had 12,300 residents (*9*,*12*,*13*,*15*,*16*).

**Table 3:** Incidence and fatality rates in a historical assessment and mapping of human plague, Kazakhstan, 1926–2003

Year	% Cases*	Incidence rate†	Case-fatality rate, %
1926	12.2	56.1	93.0
1927	1.77	8.13	70.0
1929	22.3	4.56	95.0
1945	30.8	5.3	73.0
1947	3.36	0.17	79.0
1948	11.86	0.58	72.0
1955	1.95	0.34	36.0
1956	0.35	0.08	50.0
1958	0.88	0.20	20.0
1959	0.35	0.03	50.0
1961	0.18	0.04	100.0
1964	1.95	0.34	27.0
1966	1.95	0.15	45.0
1967	2.3	0.22	15.0
1968	0.18	0.04	100.0
1969	0.35	0.06	50.0
1971	0.71	0.08	0.00
1972	0.18	0.02	100.0
1973	0.18	0.05	100.0
1974	0.35	0.10	50.0
1975	0.35	0.09	100.0
1979	0.18	0.02	0.00
1986	0.18	0.03	100.0
1988	0.18	0.03	100.0
1989	0.35	0.01	50.0
1990	0.71	0.04	50.0
1991	0.18	0.02	0.0
1992	0.18	0.02	0.0
1993	0,53	0.02	33.0
1997	0.18	0.02	0.0
1999	1.59	0.07	22.0
2001	0.35	0.03	50.0
2002	0.18	0.02	0.0
2003	0.71	0.04	25.0

*Values indicate percentage of cases recorded during the study period. †Reported as cases per 10,000 population.

We analyzed the number of outbreaks and percentage of affected population during the study period. We found that that 31 outbreaks were registered in the Mangyshlak desert NPF, accounting for 13.4% of all plague cases during the study period. In the Priaralie Karakum NPF, 17 foci and 8.8% of plague cases were registered. In the Ural-Emba desert NPF, 11 foci with 2.7% of cases were reported. In the Priustyurt desert NPF, 6 outbreaks occurred and 1.8% of the population was infected. In the Kyzylkum desert NPF, plague infected 1.1% of the population during 5 outbreaks. In the Ustyurt desert NPF, 4 outbreaks occurred, and plague infected 8.1% of the population. In the North-Priaral Desert NPF, 4 outbreaks occurred, and 31.7% of the population was infected. The Volga-Ural sandy NPF only had 1 outbreak, in which 0.2% of the population was infected. One major outbreak occurred in the Iliysk intermountain NPF, where plague infected 22.3% of the population. Two outbreaks occurred in the Pribalkhash desert NPF, where plague affected 9.9% of the population. 

**Figure 10:**
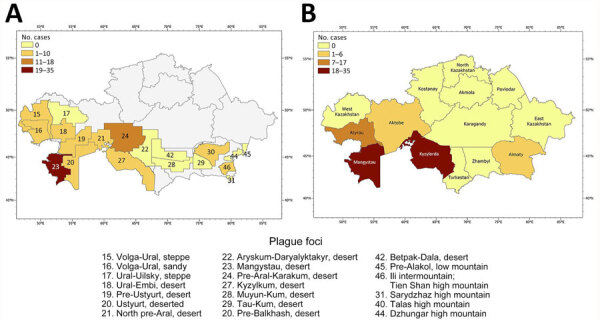
Human plague outbreaks in a historical assessment and mapping of human plague, Kazakhstan, 1926–2003. A) Number of plague outbreaks per natural plague foci; B) number of outbreaks by region.

Cases of plague among humans were registered in places with stable epizootic plague activity (Figure 10, panels A, B). Mangystau oblast had the most outbreaks, and Almaty and Aktobe oblasts had the fewest (Figure 10).

## Discussion

We analyzed 82 human plague outbreaks that occurred within 10 natural plague foci in Kazakhstan. Although human-to-human plague transmission occurred, we observed that a higher percentage of persons came in contact with *Y. pestis* through flea bites, mainly during summer and fall, and that human cases were associated with local epizootics of plague among wild rodents. Human-to-human transmission has been observed in large and small outbreaks. Other modes of infection were also observed, including cutting or skinning of animals. 

We found that camels played a major role as a source of human infections (Table 4). In summer (July and August), camels come in contact with *Y. pestis* during active plague epizootics among rodents. In September and October, camels infected during plague epizootics in summer could transmit *Y. pestis* to humans during butchering. Camels were infected by various flea vectors, depending on the season: *Xenopsylla* spp. fleas during November, December, and January, and fall flea vector fleas *Coptopsylla* spp. and *Ceratophyllus* spp. in September or October (*9*–*11*).

**Table 4:** Month and infection route in a historical assessment and mapping of human plague, Kazakhstan, 1926–2003*

Infection route	Jan	Apr	May	Jun	Jul	Aug	Sep	Oct	Nov	Dec
Human-to-human	7.43	0	0	0.18	0.53	16.81	14.69	6.90	22.30	2.12
Flea bite	0.18	0	0.88	2.65	1.59	2.30	0.53	1.59	2.48	0.35
Animal butchering or skinning	0	0	0	0.35	0	0.53	0	0	0.71	0
Rodent bite	0	0.18	0	0	0	0	0	0	0	0
Camel butchering or skinning	0.18	0	0	0	1.41	7.43	0.53	0.18	1.24	1.41
Aerosol	0	0	0	0	0.53	0	0	0	0	0
Unknown	0	0	0	0	0	1.81	0	0	0	0
Total	7.81	0.18	0.88	3.18	4.07	28.85	15.75	8.67	26.72	3.89

*Values indicate percentage of cases recorded during the study period.

We noted persons 0–19 years of age contracted plague while working in the fields, herding livestock, playing in a plague zone, and hunting with falcons and golden eagles. Adults 20–59 years of age were infected with *Y. pestis* while grazing animals, slaughtering camels, hunting, harvesting fox and hare skins, working in the fields, and other outdoor activities. Persons >60 years of age mainly were infected while helping in slaughtering animals and working in the fields.

When analyzing cases by age groups and sex, we found that, among age groups 15–19, 25–29, 50–54, and 55–59 years, cases were mostly among men and boys, who were involved in herding animals and slaughtering and skinning sick camels and wild animals. However, we noted more girls and women were infected with plague in other age groups, except in the 10–14-year age group, where the number of cases were equal among girls and boys. More (56%) girls and women were infected with plague during the major epidemics that occurred in 1945, 1947, and 1948; many were infected while cutting meat from camels or wild animals or during daily work in gardens and fields.

During the analyzed period, 70.1% of plague cases were confirmed by bacteriologic methods, 1.4% by serology after those diagnostic methods were added to surveillance efforts in 1967, and 4.1% by clinical diagnosis; 2.1% of cases had no confirmatory diagnostic data available. According to the records, some patients used antibiotic drugs before samples were collected, and no bacteriologic confirmation was made. Indirect hemagglutination reaction serologic method was used for confirmation in 2% of cases. 

The IR for plague was high in the early 20th Century and then decreased, after which the incidence plummeted as a result of plague monitoring and control programs and the use of antimicrobial drugs, insecticides, and vaccine prophylaxis (*4*,*14*). During 1926–2003, IR was 0.01–56.1/10,000 population. The mortality rate was high in almost all periods studied. For example, in the 1945 outbreak in Kyzylorda oblast, the mortality rate was 73%. In the 1920s, the mortality rate was high because treatment and prophylaxis measures were limited. In subsequent years, high mortality rates were associated with delayed treatment, incorrect and late plague diagnoses, presence of concomitant chronic diseases, late referral of patients to doctors, and remote location of patients.

Of note, study data from 2021 suggest that the area of natural foci with gopher-type *Y. pestis* Medievalis biovar 2.MED strain circulation is 221,347 km^2^ and that the *Y. pestis* sandstone-type has a 1,728,676-km^2^ range (*17*). Thus, *Y. pestis* strains of the phylogenetic branch of the Medievalis biovar 2.MED1 may have been the cause of the 1945 outbreak and 2.MED0 might have been the cause of plague outbreaks in the 1920s.

Natural plague foci in Kazakhstan vary in terms of occupied area, activity of epizootic process manifestation, and studied biocenotic and spatial structure, as well as varying risks for epidemiologic complications. On the basis of our findings, we included 139 sectors in the group with a very high degree of potential epidemic hazard, 375 sectors with high, 989 sectors with medium, 7,127 sectors with low, and 5,991 sectors with very low degree of potential epidemic hazard (*18*).

Considering the current epizootic plague situation, plague control stations and other medical and prophylactic organizations of Kazakhstan annually carry out the necessary sanitary, and preventive measures. In addition, the country conducts special plague mitigation measures, primarily epizootologic surveillance of focal areas, vaccination of humans and camels, village disinfection and deratization, creation of protective zones by field disinfection around settlements, and sanitary and educational work. The sufficient quantity and timeliness of prophylactic measures in Kazakhstan has reduced the risk for human and camel *Y. pestis* infection and absence of plague since 2003 (*18*). 

The study faced difficulties in reconstructing each plague case because the cases were recorded in different sources and some sources did not have complete data. In addition, the historical records omitted some data on patients’ sex, age, occupation, laboratory data, and clinical confirmation of cases. Those limitations may result in underestimation of incidence and fatality rates and may affect our ability to analyze demographic patterns accurately. Consequently, the study’s findings may not fully capture the scope or severity of the epidemic, potentially underrepresenting certain affected groups or trends.

## Conclusions

In Kazakhstan, from 1926 through 2003, plague cases were registered for 565 persons in 82 plague foci. Most outbreaks occurred during 1926–1948, before antibiotics and prophylactic measures were introduced in plague-endemic areas, after which the number of cases decreased. All cases were registered in plague areas of a 1,007,350-km^2^ area overlapping with stable plague epizootics. 

In summary, even though the last case was registered in 2003, plague is still relevant in Kazakhstan; active plague epizootics are observed among wild rodents in plague-endemic areas, and local plague institutions annually isolate *Y. pestis* from animals and flea vectors. Thus, to aid early warning and decision support for adequate treatment, up-to-date descriptive analyses are needed to curb effects of plague on the human population of Kazakhstan.
